# *Epichloë* endophyte interacts with saline-alkali stress to alter root phosphorus-solubilizing fungal and bacterial communities in tall fescue

**DOI:** 10.3389/fmicb.2022.1027428

**Published:** 2022-12-22

**Authors:** Hui Liu, Huimin Tang, Xiaozhen Ni, Jiazhen Zhang, Xi Zhang

**Affiliations:** College of Life Sciences, Dezhou University, Dezhou, China

**Keywords:** phosphorus solubilizing fungal (PSF) diversity, phosphorus solubilizing bacterial (PSB) diversity, soil available phosphorus, *Epichloë* endophyte, saline-alkali stress, tall fescue

## Abstract

*Epichloë* endophytes, present in aboveground tissues, modify belowground microbial community. This study was conducted to investigate endophyte (*Epichloë coenophialum*) associated with tall fescue (*Lolium arundinaceum*) interacted with an altered saline-alkali stress (0, 200 and 400 mmol/l) to affect the belowground phosphorus solubilizing microorganisms including phosphorus solubilizing fungi (PSF) and bacteria (PSB). We found that a significant interaction between *E. coenophialum* and saline-alkali stress occurred in the diversity and composition of PSF in tall fescue roots. Under saline-alkali stress conditions (200 and 400 mmol/l), *E. coenophialum* significantly increased the PSF diversity and altered its composition in the roots, decreasing the relative abundance of dominant *Cladosporium* and increasing the relative abundance of *Fusarium.* However, there was no significant interaction between *E. coenophialum* and saline-alkali stress on the PSB diversity in tall fescue roots. *E. coenophialum* significantly reduced the diversity of PSB in the roots, and *E. coenophialum* effects did not depend on the saline-alkali stress treatment. Structural equation modeling (SEM) showed that *E. coenophialum* presence increased soil available phosphorus concentration under saline-alkali stress primarily by affecting PSF diversity instead of the diversity and composition of PSB.

## 1. Introduction

Plant tissues form a wide variety of symbiotic associations with above and belowground microorganisms, whose interactions range from parasitism to mutualism (Liu et al., 2021). An example of aboveground plant microorganisms that often occur worldwide between cool-season grasses such as perennial ryegrass and tall fescue are the fungal endophytes (family Clavicipitaceae, genus *Epichloë*; Leuchtmann et al., 2014; Slaughter et al., 2018). In the grass-*Epichloë* endophytes symbiotic relationship, the grass provides the endophytes with nutrients and shelter, and in exchange, the endophytes promote grass growth and confer protection against abiotic such as drought and saline-alkali and biotic such as herbivores and foliar pathogens stressors (Chen et al., 2017; Liu et al., 2017, 2022a; Qin et al., 2019; Li et al., 2020; Yang et al., 2020). Although the consequences of *Epichloë* endophyte infection on the growth and resistance of host grasses have been well documented, to our knowledge, only a few papers have measured the impact of aboveground *Epichloë* endophytes on belowground components, especially on microorganisms (Arrieta et al., 2015; Vignale et al., 2016; Zhong et al., 2018; Liu et al., 2020, 2022b; Mahmud K. et al., 2021).

Roots are colonized, both internally and externally, by a wide range of root-associated microorganisms (Keim et al., 2014). Plant root secretes root exudates such as carbohydrates, proteins, secondary metabolites, etc. enhancing arbuscular mycorrhizal fungi (AMF) and plant growth-promoting rhizobacteria (PGPR) root colonization either directly or through regulating their gene expression (Singh P. et al., 2022; Upadhyay et al., 2022a). These root-associated microorganisms promote plant growth through root-hair proliferation, enhancing soil fertility; increase in nitrogen fixation ability; enhanced leaf surface area; improvement in vigor and biomass; increased indigenous plant hormones levels; and most importantly, by improving nutrient use efficiency (Fan et al., 2020; Mahmud A. A. et al., 2021; Singh R. K. et al., 2022). The most studied microorganisms that are affected by the *Epichloë* endophytes are AMF, and the results have shown that *Epichloë* endophytes can change the colonization, diversity and community of AMF (Vignale et al., 2016; Liu et al., 2020, 2022a,b; Terlizzi et al., 2022).

In addition to AMF, another important beneficial microbial group are phosphorus solubilizing microorganisms (PSMs) including phosphorus solubilizing fungi (PSF) and bacteria (PSB), and they may contribute to plant nutrition by increasing the pool of phosphorus (P) through the hydrolysis of organic P compounds and insoluble inorganic P sources, thereby making P available for plant assimilation (Arrieta et al., 2015; Kalayu, 2019; Yin et al., 2020). The mechanisms of organic and inorganic P solubilization made by PSMs involve synthesis of metabolites such as organic acids, chelation of cations, as well as synthesis of phosphatase enzymes that hydrolyse organic P forms to inorganic P (Rashid et al., 2004; Jarosch et al., 2019; Kalayu, 2019). Both PSF and PSB exhibit P solubilization (Bolo et al., 2021). The most powerful PSF include some species of the genera *Aspergillus* and *Penicillium* (Arrieta et al., 2015; Kalayu, 2019; Bolo et al., 2021). PSB in genera *Pseudomonas*, *Bacilli*, *Rhizobium* and *Agro-bacterium* also have P solubilizing abilities (Bolo et al., 2021).

To our knowledge, there are just a few studies that have evaluated the effects of *Epichloë* endophytes on PSMs. Arrieta et al. (2015) only focusing on PSF have demonstrated that *Epichloë* endophytes increase the diversity of PSF in rhizosphere soil of *Bromus auleticus*. In addition, many studies have emphasized that the key role of environmental context on the interaction of aboveground *Epichloë*-belowgroud microorganisms (Ding et al., 2021; Liu et al., 2022b). For example, Ding et al. (2021) in tall fescue found that *Epichloë* endophytes had a greater effect on rhizosphere general fungi under P limiting conditions. Liu et al. (2022b) showed that the effects of *Epichloë* endophytes on fungal and bacterial diversity occurred in 200 mmol/l saline-alkali stress but not in either non-saline-alkali or other saline-alkali stress conditions.

Soil salinization is a severe agronomical, ecological, and socioeco-nomic problem in most arid and semiarid regions worldwide (Munns and Tester, 2008; Estrada et al., 2013). Extensive fertilization, desertification processes, urbanization, uncon-trolled irrigation practices, etc. are the main factors triggering salinity (Abd-Alla et al., 2019; Upadhyay and Chauhan, 2022b). The salinization of the soil is increasing and more than 50% of the global arable land is predicted to be salinized by the year 2050 (Butcher et al., 2016). The belowground microbial communities were commonly influenced by saline-alkali conditions (Liu et al., 2021; Che et al., 2022). In this study, we focused on the effects of saline-alkali stress and *Epichloë* endophytes in the aboveground parts of tall fescue on belowground processes including PSF and PSB. Therefore, we hypothesize that (1) *Epichloë* endophyte will alter the belowground PSF and PSB diversity and community composition, and (2) that this effect will change with the level of saline-alkali stress.

## 2. Materials and methods

### 2.1. Plant material

The endophyte-infected (EI) and endophyte-free (EF) tall fescue seeds were provided by Professor Anzhi Ren at Nankai University. EI seeds were naturally infected with *Epichloë coenophialum* (Morganjones and Gams, 1982; Leuchtmann et al., 2014), and infection by *E. coenophialum* was verified by staining and microscopic analysis using aniline blue (Latch and Christensen, 1985) and by isolation of the fungus from plant leaf sheaths on potato dextrose agar (PDA) in Petri dishes. EF seeds were obtained by storing EI seeds at room temperature for 1 year to inactivate the endophyte.

### 2.2. Experimental design and harvest

Tall fescue plants were used in the experiments following a combined factorial design with two factors: (1) plants infected (EI) or not infected (EF) with the endophyte *E. coenophialum*, (2) plants grown under non-saline-alkali stress or saline-alkali stress conditions including two stress levels. Combinations of the two factors gave six different treatments (2 endophyte infection status × 3 saline-alkali stress levels) with a total of 30 pots (five replicates per treatment).

Seeds of tall fescue were sown at the soil surface at the rate of 20 seeds per pot in separate plastic pots (18 cm diameter × 16 cm height), each filled with 1.2 kg of normal soil. After germination (at 7 days), the plants were thinned to 12 uniform plants per pot. The growth conditions in the greenhouse at the College of Life Sciences, Dezhou University, China were as follows: 19–25°C, 40–50% relative humidity, and natural daylight.

After 6 weeks, seedlings were treated solution with or without saline-alkali (molar ratio of NaCl: Na_2_SO_4_: NaHCO_3_: Na_2_CO_3_ = 9: 1: 1: 9, simulating mixed saline-alkali stress conditions according to the ion composition of saline-alkali soil in Northeast China). The EI and EF seedlings were subjected to the following saline-alkali stress: 200 and 400 mM. The saline-alkali levels determined in the experiment matched the range of natural environmental conditions without leading to extremely high mortality (Liu et al., 2022b). To avoid osmotic shock, 300 ml of each saline-alkali solution was gradually introduced by successively adding 100 ml every 2 days; an equal amount of distilled water was added to the control pots. The soil water content was controlled with a soil moisture probe (ECH_2_O Check; Decagon Devices, Pullman, WA, United States) every day, and the lost water was supplemented with distilled water.

After 60 days exposure to saline-alkali stress, plant roots and rhizosphere soil samples were collected. The roots were carefully rinsed with distilled water and stored at −80°C before DNA extraction. The rhizosphere soil was collected by brushing the soil from the root surface with a sterilized soft-bristled paintbrush for available phosphorus determination.

### 2.3. Soil available phosphorus

The soil samples were air-dried and passed through a sieve (2 mm). The soil available phosphorus concentration was calculated by shaking the soil samples with NaHCO_3_ solution (pH 8.5) and then colorimetrically analyzing the samples using the molybdenum blue method (Robertson et al., 1999).

### 2.4. DNA extraction, sequencing, and microbial community analysis

To analyze the composition of PSF and PSB communities in the root samples with different saline-alkali stress, microbial DNA from each sample was extracted by using the FastDNA® SPIN for soil kit (MP Biomedicals, Santa Ana, CA, United States). Extracted DNA was amplified using a ITS1F (5’-CTTGGTCATTTAGAGGAAGTAA-3′) and ITS2R (5’-GCTGCGTTCTTCATCGATGC-3′) universal primer set targeting the ITS1 region of the fungi as well as a 799F (5’-AACMGGATTAGATACCCKG-3′) and 1193R (5’-ACGTCATCCCCACCTTCC-3′) universal primer set targeting the V5–V7 region of the bacterial 16S rRNA. PCR products were purified and sequenced on an Illumina MiSeq platform (Illumina, San Diego, United States) by the standard protocols of Majorbio Bio-Pharm Technology Co. Ltd. (Shanghai, China).

The sequences among all reads were used to define operational taxonomic units (OTUs) using UPARSE (version 7.1^1^) with 97% sequence similarity. The sequences of all other samples were subsampled with the minimum number of reads to compare different samples at the same sequencing level (Fang et al., 2018). Final OTUs were taxonomically classified using the RDP Classifier algorithm^2^ against the Unite7.2 ITS database and the Silva132 16S rRNA database at a confidence threshold of 70%.

### 2.5. Statistical analysis

The effects of the saline-alkali stress and *Epichloë* endophyte infection on the phosphorus solubilizing fungal (PSF) diversity, phosphorus solubilizing bacterial (PSB) diversity and soil available phosphorus (AP) concentration were analyzed using two-factor analysis of variance (ANOVA) with SPSS 20.0 (SPSS Inc., Chicago, IL, United States). When a significant effect was detected, the differences between the means of different treatments were determined using Duncan’s multiple-range tests at *p* = 0.05. PSF and PSB diversity were estimated as the effective number of species using the exponential of Shannon diversity index. Variation in the PSF and PSB community composition was visualized using non-metric multidimensional scaling (NMDS) ordination, using the metaMDS function in the VEGAN package. Structural equation modeling (SEM) was fitted to our data using SPSS Amos 21.0 to identify potential causal relationships between explanatory variables (PSF and PSB diversity and community composition in roots) and soil AP concentration.

## 3. Results

### 3.1. Soil available phosphorus concentration

**Figure 1:**
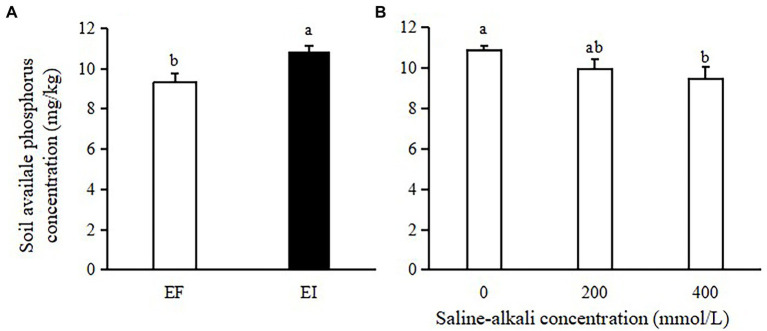
Effects of *Epichloë* endophyte **(A)** and saline-alkali stress **(B)** on soil available phosphorus concentration. Values are means ± SE. Different letters denote means that are significantly different (*p* < 0.05).

The soil available phosphorus (AP) concentration was significantly affected by saline-alkali stress (*F* = 3.398, *p =* 0.050) and *Epichloë* endophyte infection (*F* = 11.645, *p =* 0.002). The soil AP concentration was increased by *Epichloë* endophyte infection (16%, Figure 1A) but decreased by saline-alkali stress (11% on average, Figure 1B).

### 3.2. PSF and PSB diversity

**Figure 2:**
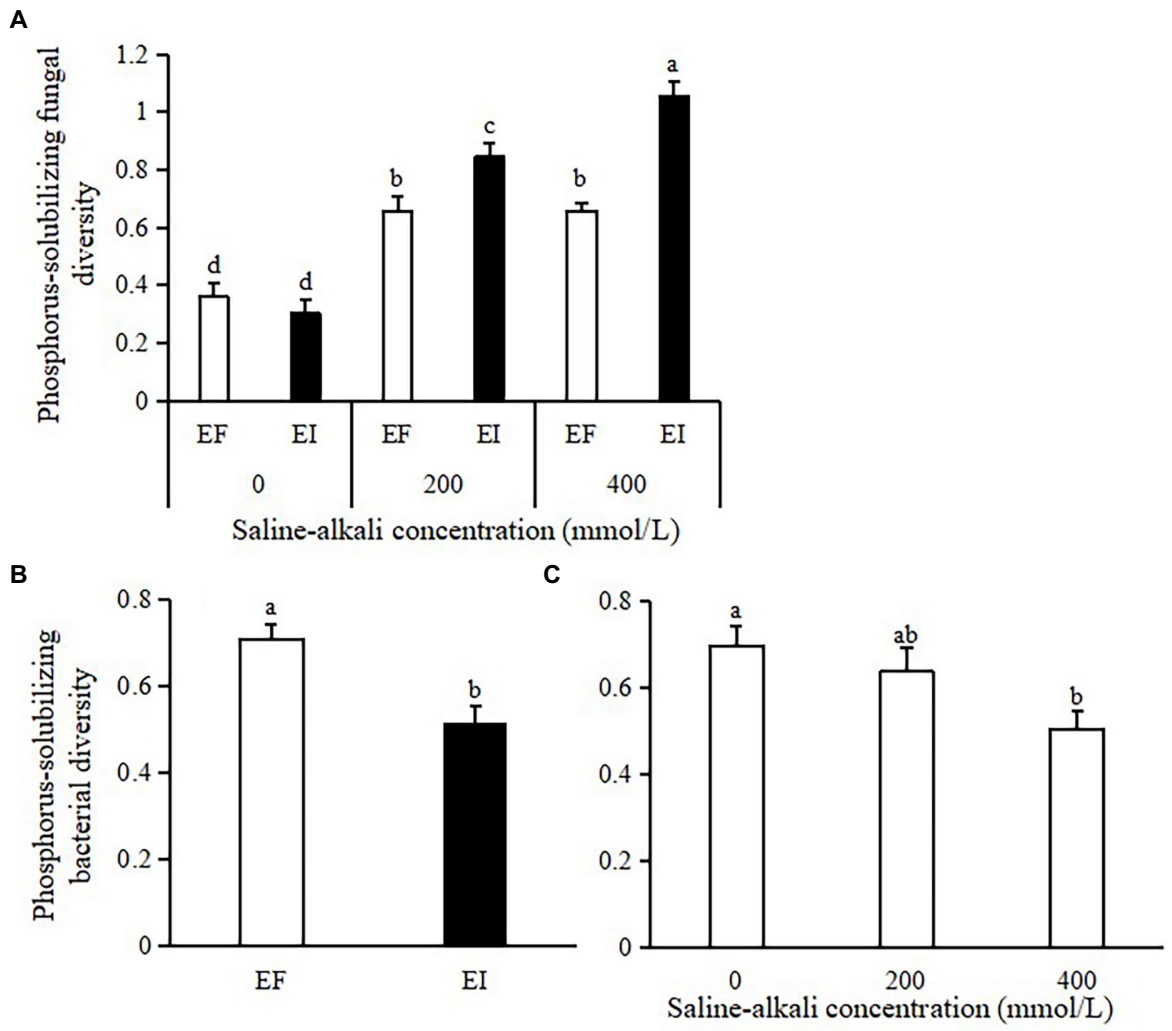
Effects of the interaction between *Epichloë* endophyte and saline-alkali stress on phosphorus solubilizing fungal (PSF) diversity in tall fescue roots **(A)**, and effects of *Epichloë* endophyte **(B)** and saline-alkali stress **(C)** on phosphorus solubilizing bacterial (PSB) diversity in tall fescue roots. Different letters denote means that are significantly different (*p* < 0.05).

The PSF diversity was significantly affected by saline-alkali stress, *Epichloë* endophyte infection and their interactions (*F* = 12.272, *p* < 0.001). Under non-stress (0 mM) conditions, EI and EF tall fescue had similar PSF diversity. Saline-alkali stress significantly increased PSF diversity (*F* = 73.249, *p* < 0.001). *Epichloë* endophyte infection significantly increased the PSF diversity of tall fescue by 29 and 60% in the 200 and 400 mM saline-alkali stress conditions, respectively, (Figure 2A). The PSB diversity of tall fescue was significantly affected by the main effects of *Epichloë* endophyte (*F* = 22.380, *p* < 0.001), with *Epichloë* endophyte infection significantly decreasing the PSB diversity of tall fescue by 27% (Figure 2B). Saline-alkali stress also significantly decreased the PSB diversity (18% on average; *F* = 7.823, *p =* 0.002; Figure 2C).

### 3.3. PSF and PSB community composition

**Figure 3:**
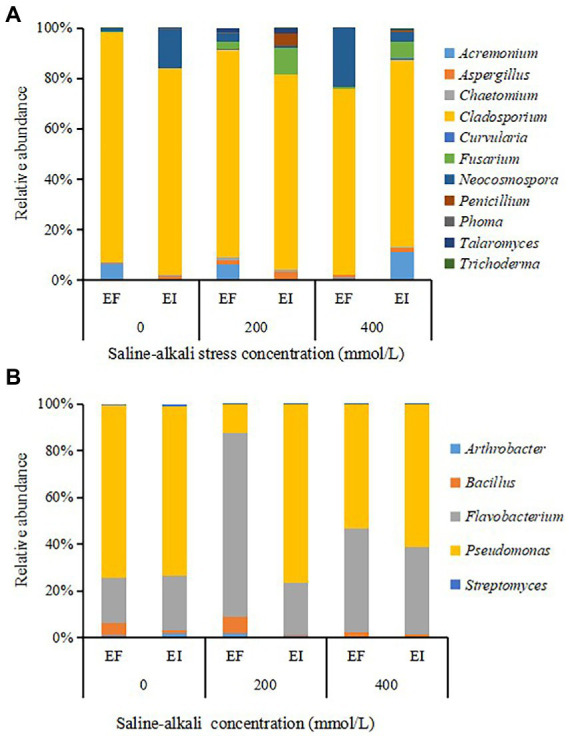
The composition of different genera of phosphorus solubilizing fungi (PSF) **(A)** and phosphorus solubilizing bacteria (PSB) **(B)** in roots of tall fescue with (EI) and without (EF) *Epichloë* endophyte under saline-alkali stress.

The 11 PSF genera covered by OTUs were *Acremonium*, *Aspergillus*, *Chaetomium*, *Cladosporium*, *Curvularia*, *Fusarium*, *Neocosmospora*, *Penicillium*, *Phoma*, *Talaromyces* and *Trichoderma*. *Cladosporium* was the dominant genus in all treatments, and its proportion ranged from 73.7 to 91.2%. *Epichloë* endophyte infection decreased the relative abundance of dominant *Cladosporium* and increased the relative abundance of *Fusarium* under saline-alkali stress conditions (Figure 3A). The PSB community comprised members of the genera *Arthrobacter*, *Bacillus*, *Flavobacterium*, *Pseudomonas* and *Streptomyces*. All treatments were dominated by the *Flavobacterium* (37.5% on average) and *Pseudomonas* (58.1% on average; Figure 3B).

**Figure 4:**
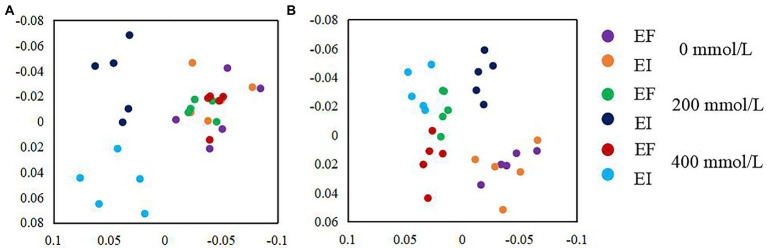
Non-metric multidimensional scaling (NMDS) ordination of PSF **(A)** and PSB **(B)** community composition in roots of tall fescue with (EI) and without (EF) *Epichloë* endophyte under saline-alkali stress.

NMDS based on the relative abundance of OTUs clustering by saline-alkali stress and *Epichloë* endophyte revealed that there was a significant effect of the interaction between saline-alkali stress and *Epichloë* endophyte on the community composition of PSF and PSB. Under non-stress (0 mM) conditions, there was not a significant difference either in PSF or PSB communities between EF and EI tall fescue. However, a clear separation in the PSF and PSB communities between EI and EF tall fescue was observed in 200 and 400 mM saline-alkali stress conditions (Figures 4A,B).

### 3.4. Relationship between PSF, PSB diversity, community composition and soil available phosphorus

**Figure 5:**
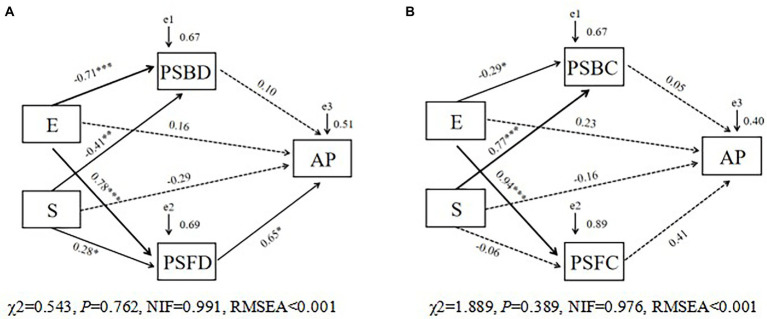
The structural equation model (SEM) showing the hypothesized causal relationships between explanatory factors [**(A)** PSF and PSB diversity; **(B)**, PSF and PSB community composition] and available phosphorus concentration in rhizosphere soil of tall fescue under saline-alkali stress. The width of arrows indicates the strength of the standardized path coefficient (***, *p* < 0.001; **, *p* < 0.01; *, *p* < 0.05). The e-values represent residuals. E, endophyte infection status; S, saline-alkali stress; PSFD, phosphorus solubilizing fungal diversity; PSBD, phosphorus solubilizing bacterial diversity; PSFC, phosphorus solubilizing fungal community composition; PSBC, phosphorus solubilizing bacterial community composition.

We used SEM to assess the extent of direct and indirect effects of saline-alkali stress (S) and *Epichloë* endophyte infection (E) on the soil available phosphorus (AP) concentration of the tall fescue (Figures 5A,B). Saline-alkali stress and *Epichloë* endophyte infection had no direct effects on the soil AP concentration, but these two treatments increased the soil AP concentration by indirectly increasing the PSF diversity (PSFD; Figure 5A) but not by affecting PSB community diversity or composition (PSFC; Figure 5B).

## 4. Discussion

Soil salinization is detrimental to plant growth and yield in agroecosystems worldwide. One possible improvement strategy is to explore the potential of associated salt-tolerant microorganism to confer saline-alkali stress tolerance to plants (Moreira et al., 2020; Yang et al., 2020). The suitable root-associated microorganisms and components of root exudate interplay against stress (Singh P. et al., 2022; Upadhyay et al., 2022a). In our study, we found that the diversity of the PSF of saline-alkali stress conditions was higher than that in the non-saline-alkali stress conditions; however, the diversity of PSB showed the opposite direction, with higher diversity in non-saline-alkali stress than that in saline-alkali stress conditions. Therefore, compared with PSB, most PSF were saline-alkali tolerant. Among PSF, *Cladosporium* had the largest relative abundance and therefore an absolute advantage. The relative abundance of *Cladosporium* decreased with the increase of saline-alkali stress.

The *Epichloë* endophytes existing in the aboveground part of the host grass have been demonstrated that can produce effects on root microorganisms, and they can change the impact of environment condition on root microorganisms (Bell-Dereske et al., 2017; Slaughter et al., 2018; Zhong et al., 2021; Liu et al., 2022b). Bell-Dereske et al. (2017) in *Ammophila breviligulata* showed that with *Epichloë amarillans* infected, the diversity of root-associated bacteria declined with higher soil moisture, whereas in its absence, bacterial diversity increased with higher soil moisture. Slaughter et al. (2018) in tall fescue found that *E. coenophiala* significantly decreased the rate of AMF arbuscule formation in treatments without added precipitation, but had no significant effect in added precipitation treatments. Zhong et al. (2021) in *Achnatherum inebrians* showed that *Epichloë gansusensis* increased root-associated AMF diversity under drought conditions, while decreasing diversity under the water addition treatment. Liu et al. (2022b) demonstrated that the effects of *Epichloë* endophyte infection on AMF diversity shifted from neutral in non-saline-alkali stress to positive in 200 and 400 mmol/l saline-alkali stress. Little is known about the *Epichloë*-PSMs interaction and there is even less knowledge about the interaction *Epichloë*-PSMs-saline-alkali stress. Arrieta et al. (2015) in *B. auleticus* showed that *Epichloë pampeana* increased the diversity of PSF. In the present study, a synergistic effect occurred between *E. coenophiala* and saline-alkali stress on the diversity of PSF in tall fescue roots, and the PSF diversity of tall fescue roots infected with *E. coenophiala* under saline-alkali stress was significantly higher than that of tall fescue roots infected with *E. coenophiala* alone or treated with saline-alkali alone. In addition, *E. coenophiala* presence altered the relative abundance of several PSF groups, including decreasing the relative abundance of dominant *Cladosporium* and increasing the relative abundance of *Fusarium*. *Fusarium* has been previously recorded able to solubilize P. NMDS ordination revealed that a clear separation in the PSF communities between EI and EF plant roots was observed in 200 and 400 mmol/l saline-alkali stress conditions.

In contrast to PSF, there was no interaction between *E. coenophiala* and saline-alkali stress on the diversity of PSB in tall fescue roots. *E. coenophiala* decreased the diversity of PSB in tall fescue roots regardless of saline-alkali stress level. However, a significant effect of the interaction between *E. coenophiala* and saline-alkali stress on the composition of PSB communities was observed. There was a clear separation between the PSB communities due to *E. coenophiala* presence that occurred both under 200 and 400 mmol/l saline-alkali stress conditions, whereas *E. coenophiala* presence had no obvious effect on the PSB community composition under non-stress conditions.

Phosphorus is an essential element for plant development and growth, making up about 0.2% of plant dry weight (Smith et al., 2011). Plants obtain phosphorus from soil solution in the form of phosphorus anion. However, phosphate anions react easily and are fixed by precipitation with cations such as Ca^2+^, Mg^2+^, Fe^3+^ and Al^3+^ under saline-alkali stress conditions due to high pH, becoming highly insoluble phosphate that is not available to plants (Machiavelli and Khurana, 2013; Singh P. et al., 2022; Upadhyay and Chauhan, 2022b). Rangseekaew et al. (2021) showed that rhizospheric bacteria promoted plants growth under NaCl stress resulted from the production of many plant-growth promoting attributes such as siderophore production, indole-3-acetic acid, and phosphate solubilization. In our study, saline-alkali stress and the presence of the *E. coenophiala* brought changes to the soil available phosphorus (AP), and a close association was also observed between the belowground phosphorus solubilizing microorganisms and soil AP. SEM results revealed that *E. coenophiala* presence had no direct effects on the soil AP, but increased the soil AP directly by increasing the diversity of PSF in tall fescue roots under saline-alkali stress conditions.

## 5. Conclusion

Our results demonstrated that *E. coenophiala* significantly increased the diversity of PSF and altered the community composition of PSF in tall fescue roots under saline-alkali stress conditions but did not affect those parameters mentioned above under non-stress conditions. By contrast, both *E. coenophiala* and saline-alkali stress significantly decreased the diversity of PSB in tall fescue roots. Furthermore, the positive effects of the *E. coenophiala* on PSF diversity, generate a significant increase in the phosphorus available to plants under saline-alkali stress conditions, making this a very interesting model to evaluate its impact on grasses of economic interest.

## Data availability statement

The datasets presented in this study can be found in online repositories. The names of the repository/repositories and accession number(s) can be found below: NCBI - PRJNA883018.

## Author contributions

HL designed the research. HT, XN, JZ, and XZ performed the experiments. HL and HT analyzed the data and wrote the manuscript. HL revised and polished the manuscript. All authors contributed to the article and approved the submitted version.

## Funding

This work was supported by the National Natural Science Foundation of China (32001103) and Dezhou University Science Research Foundation (2019xjrc317).

## Conflict of interest

The authors declare that the research was conducted in the absence of any commercial or financial relationships that could be construed as a potential conflict of interest.

## Publisher’s note

All claims expressed in this article are solely those of the authors and do not necessarily represent those of their affiliated organizations, or those of the publisher, the editors and the reviewers. Any product that may be evaluated in this article, or claim that may be made by its manufacturer, is not guaranteed or endorsed by the publisher.

## Footnotes

^1^ http://drive5.com/uparse/

^2^ http://rdp.cme.msu.edu/
